# External validation of HAS model in predicting mortality after emergency laparotomy: a retrospective cohort study

**DOI:** 10.1308/rcsann.2025.0021

**Published:** 2025-04-03

**Authors:** H Soliman, C Smith, J Mena, GT Yusuf, AH Helmy

**Affiliations:** King's College Hospital NHS Foundation Trust, UK

**Keywords:** Laparotomy, Mortality, Risk assessment, Sarcopenia, Perioperative care

## Abstract

**Introduction:**

We aimed to externally validate the performance of the HAS model (Hajibandeh Index, American Society of Anaesthesiologists status, and sarcopenia) in predicting mortality after emergency laparotomy. We also aimed to compare the HAS model with the Parsimonious NELA (National Emergency Laparotomy Audit) risk score.

**Methods:**

In this retrospective cohort study, we included adult patients who underwent emergency laparotomy between January 2022 and June 2023. The performance of the HAS score and the NELA score in predicting 30-day mortality was compared using receiver operating characteristic (ROC) curve analysis. We performed subgroup analysis for the following age groups: age ≥50, age ≥60, age ≥70, and age ≥80 years.

**Findings:**

We included 117 patients in this study. ROC curve analysis showed that area under the curve (AUC) of the HAS score for 30-day mortality was 0.90 (95% CI 0.83–0.95). Although the AUC of HAS score was higher than the AUC of NELA score for all patients, this was not statistically significant (0.90 vs 0.80, *p*=0.097). AUC of the HAS score was superior to NELA score in patients aged ≥50 (0.89 vs 0.75, *p*=0.040), patients aged ≥60 (0.87 vs 0.69, *p*=0.020), patients aged ≥70 (0.85 vs 0.67, *p*=0.030), and patients aged ≥80 (0.90 vs 0.66, *p*<0.001).

**Conclusions:**

The results of the current study support the external validity of the HAS model in predicting 30-day mortality after emergency laparotomy. Prospective studies with larger sample size are required.

## Introduction

Emergency laparotomy is frequently associated with a higher risk of morbidity and mortality. These procedures are typically performed under urgent or emergent conditions to address life-threatening conditions, such as bowel perforation, ischemia or severe infection. The nature of emergency laparotomies, involving critically ill patients with complex medical histories, necessitates accurate preoperative risk assessment tools to aid in clinical decision-making and patient management.^1,2^

Over recent years, the need for preoperative mortality risk assessment tools has led to the development and validation of preoperative mortality risk assessment tools, which have become increasingly popular. These tools provide valuable information that can guide surgeons and anaesthesiologists in evaluating the risks and benefits of proceeding with surgery.

A precise estimation of mortality risk is essential for several reasons. (1) Objective decision-making: surgeons and patients (or their families) need to have frank discussions about the potential outcomes of surgery. A dependable risk assessment tool offers an objective foundation for these conversations, guaranteeing that patients or their representatives are thoroughly informed. (2) Planning and resource allocation: high-risk patients may require more intensive perioperative monitoring and postoperative care. Predicting which patients are at greater risk can help in planning the appropriate level of support and resource allocation. (3) Improving patient outcomes: by identifying high-risk patients, healthcare providers can implement targeted strategies to mitigate these risks. This may include optimising medical conditions preoperatively, ensuring the availability of critical care resources and planning for postoperative rehabilitation.^2–5^

Despite the proliferation of risk assessment tools, the search for a predictive model with excellent accuracy remains ongoing. An ideal model would incorporate a comprehensive set of variables that accurately reflect the patient’s health status and the severity of their condition.

Hajibandeh *et al*^2^ recently introduced a novel mortality risk predictive model known as the HAS (Hajibandeh Index, American Society of Anaesthesiologists status, and sarcopenia) model. This model demonstrated excellent performance in terms of discrimination, classification and calibration, suggesting its potential utility in clinical practice.^2^ The HAS model comprises three primary components: (1) Hajibandeh Index (HI) – a proprietary index developed by the authors, integrating various clinical parameters to gauge the severity of the patient’s condition. (2) American Society of Anaesthesiologists (ASA) status – a commonly used classification system that evaluates the physical condition of patients before surgery. (3) Sarcopenia – this refers to the loss of skeletal muscle mass and function, which is a critical predictor of adverse outcomes in surgical patients. The inclusion of sarcopenia highlights the importance of physiological reserve in determining surgical risk.^2^

The model was derived through a comprehensive multivariable analysis that included significant predictors of mortality following emergency laparotomy. These predictors included age ≥80 years, HI, ASA status, sarcopenia, clinical frailty scale (CFS), necessity for bowel resection and the presence of intraperitoneal contamination.

The HAS model was found to outperform the National Emergency Laparotomy Audit (NELA) mortality risk score, which is widely adopted in the UK and has limitations in accuracy and comprehensiveness, prompting the need for improved models like HAS.^6^ The NELA score was updated in April 2023 to the parsimonious NELA score to improve its predictive accuracy and simplify the risk assessment process. Despite the promising results of the HAS model, the authors acknowledged the necessity of external validation.^2,6^ Validation involves testing the model on a different patient cohort to ensure that its predictive accuracy holds across various settings and populations. This step is crucial before the model can be recommended for routine clinical use. The primary aim of this study was to externally validate the performance of the HAS model in predicting mortality following emergency laparotomy. Additionally, the study seeks to compare the HAS model with the parsimonious NELA mortality risk score to determine which tool provides better predictive accuracy.

## Methods

This study used a retrospective cohort design, conducted at a general surgery department in a large district general hospital in England. Given the retrospective nature of the study, ethical approval was not required. The study was registered locally as a service evaluation project. The study included adult patients who underwent nontraumatic emergency laparotomy between January 2022 and June 2023. The principal inclusion criteria were age ≥18 years and have undergone an emergency laparotomy due to acute nontraumatic abdominal pathology. Patients were excluded if the laparotomy was performed due to trauma. Additionally, patients without a preoperative computed tomography (CT) scan were excluded because of the assessment of sarcopenia. Finally, patients without available perioperative data necessary for calculating the HAS or the parsimonious NELA score were excluded, as missing data would compromise the validity of the study.

The data collection process was comprehensive, relying on the electronic medical record system of the hospital to identify and gather relevant information for each patient including age, sex, ASA status, indication for laparotomy, the procedure performed, variables required for calculation of HAS score and NELA score, and mortality outcomes.

### Predicted mortality risk scores

The HAS predicted mortality score is calculated using the HAS Emergency Laparotomy Mortality Risk Calculator.^7^ The HAS score incorporates three primary factors: HI, ASA status and the presence of sarcopenia.^2^

(1) HI: this is derived from a formula developed by Hajibandeh *et al* ^5^ using C-reactive protein (CRP), neutrophils and lactate as nominators and albumin and lymphocytes as denominators. This combination of biomarkers helps in assessing the inflammatory and nutritional status of the patient, which are critical indicators of surgical risk.^5^

(2) ASA status: this is a widely recognised classification system that evaluates the physical status of patients before surgery. It ranges from ASA I, a normal healthy patient; ASA II, a patient with mild systemic disease; ASA III, a patient with severe systemic disease; ASA IV, a patient with a severe systemic disease that is a constant threat to life; and ASA V, a moribund patient who is not expected to survive without the operation.^8^

(3) Sarcopenia: this is assessed using the psoas muscle index (PMI). The PMI is calculated by measuring the cross-sectional area of both psoas muscles at the inferior level of the L3 vertebral body on CT scan adjusted based on the patient’s height, age and sex to account for individual variations. Sarcopenia is a significant predictor of poor surgical outcomes, particularly in older adults.

The NELA predicted mortality score is calculated using the parsimonious NELA mortality risk calculator, which considers a comprehensive range of factors as follows: (1) Demographics: age and gender. (2) Preoperative clinical factors: ASA status, preoperative laboratory tests including albumin, urea, white cell count and Glasgow coma score. (3) Vital signs: systolic blood pressure, heart rate, respiratory signs of distress. (4) Surgical factors: operative severity, peritoneal soiling, severity of malignancy and urgency of surgery.^9^

### Outcomes and statistical analyses

The primary outcome measure for evaluating these mortality risk scores was the 30-day postoperative mortality defined as mortality due to any cause occurring within 30 days after the emergency laparotomy. The data collected for analysis included demographics, clinical characteristics and outcomes. These data were summarised using either mean or median values for continuous variables and percentages for categorical variables.

To compare the predictive accuracy of the HAS and the parsimonious NELA mortality risk scores, receiver operating characteristic (ROC) curve analysis was employed. The area under the curve (AUC) was calculated for each score to quantify their discriminative power. Additionally, subgroup analyses were performed for different age groups: age ≥50, age ≥60, age ≥70 and age ≥80 years. This stratification allowed for a more detailed assessment of the predictive performance of the scores across different age brackets, which is particularly relevant given the increased surgical risks associated with older age.

All statistical tests conducted were two-tailed, with a significance level set at 95% confidence. The statistical analyses were carried out using MedCalc version 13.0, a comprehensive statistical software package designed for biomedical research.

## Results

From January 2022 to June 2023, a total of 132 patients underwent emergency laparotomy due to nontraumatic abdominal pathology. Of these, 15 patients were excluded from the study due to the unavailability of perioperative data, leaving 117 patients for the final analysis.

The median age was 74 years, with an interquartile range (IQR) of 58 to 81 years, indicating a relatively older patient population. In terms of sex, 46% (54/117) were male and 54% (63/117) were female. This near-equal sex distribution provides a balanced perspective on the outcomes across sexes.

In terms of preoperative physical status, assessed using the ASA classification, the distribution was as follows: ASA I: 9 patients (8%), ASA II: 43 patients (37%), ASA III: 60 patients (51%) and ASA IV: 5 patients (4%). The actual observed risk of 30-day mortality in this cohort was 12%, with 14 out of the 117 patients succumbing within this period. The mean predicted mortality risk according to the HAS score was 12%, with a 95% confidence interval (CI) ranging from 8% to 16%, while the mean predicted mortality risk as per the NELA score was slightly higher at 14%, with a 95% CI of 12% to 17%.

These predicted risks are closely aligned with the actual observed mortality rate, suggesting that both models provide reasonably accurate predictions within the studied cohort. The baseline characteristics of the patients included are summarised in Table 1.

**Table 1:** Baseline characteristics of included patients

Number of patients	117
Age, median (IQR)	74 (58–81)
Age ≥ 50	95 (81%)
Age ≥ 60	82 (70%)
Age ≥ 70	67 (57%)
Age ≥ 80	36 (31%)
Male, *n* (%)	54 (46%)
Female, *n* (%)	63 (54%)
ASA grade, *n* (%)	
I	9 (8%)
II	43 (37%)
III	60 (51%)
IV	5 (4%)
Actual 30-day mortality, *n* (%)	14 (12%)
HAS predicted mortality risk, mean (95% CI)	12% (8–16)
NELA predicted mortality risk, mean (95% CI)	14% (12–17)

ASA = American Society of Anesthesiologists; CI = confidence interval; HAS = Hajibandeh Index, American Society of Anaesthesiologists status, and sarcopenia; IQR = interquartile range; NELA = National Emergency Laparotomy Audit.

### Performance of HAS score versus NELA score for 30-day mortality

**Figure 1:**
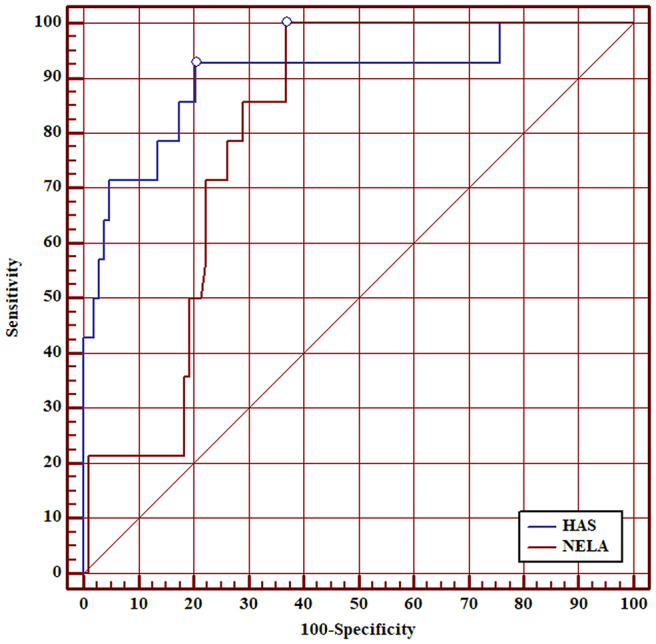
ROC curve for comparison between HAS score and NELA score in predicting 30-day mortality in all patients. HAS = Hajibandeh Index, American Society of Anaesthesiologists status, and sarcopenia; NELA = National Emergency Laparotomy Audit; ROC = receiver operating characteristic.

**Figure 2:**
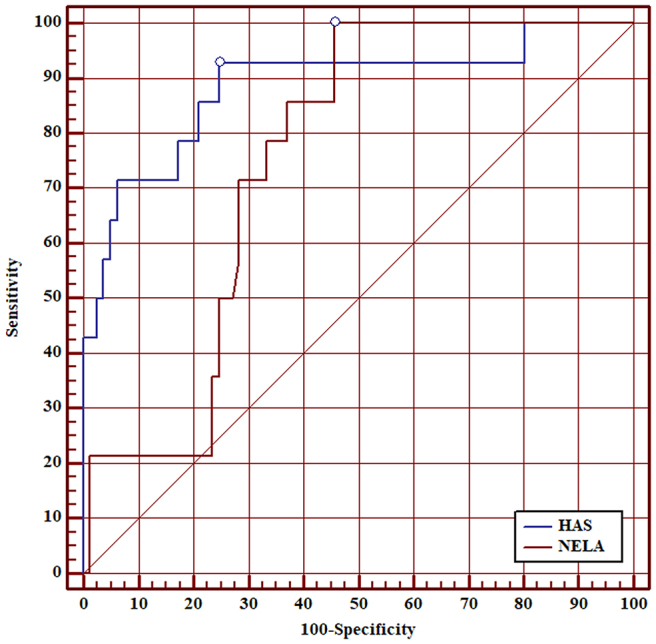
ROC curve for comparison between HAS score and NELA score in predicting 30-day mortality in: patients aged ≥50 years. HAS = Hajibandeh Index, American Society of Anaesthesiologists status, and sarcopenia; NELA = National Emergency Laparotomy Audit; ROC = receiver operating characteristic.

**Figure 3:**
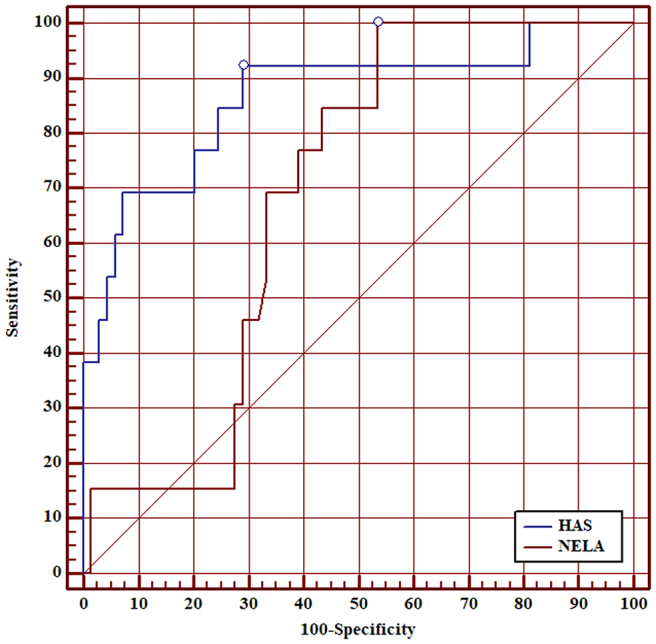
ROC curve for comparison between HAS score and NELA score in predicting 30-day mortality in patients aged ≥60 years. HAS = Hajibandeh Index, American Society of Anaesthesiologists status, and sarcopenia; NELA = National Emergency Laparotomy Audit; ROC = receiver operating characteristic.

**Figure 4:**
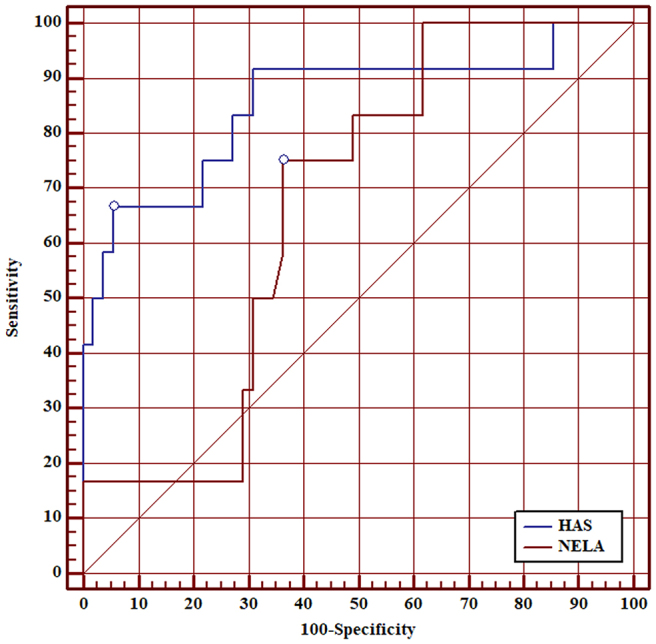
ROC curve for comparison between HAS score and NELA score in predicting 30-day mortality in patients aged ≥70 years. HAS = Hajibandeh Index, American Society of Anaesthesiologists status, and sarcopenia; NELA = National Emergency Laparotomy Audit; ROC = receiver operating characteristic.

**Figure 5:**
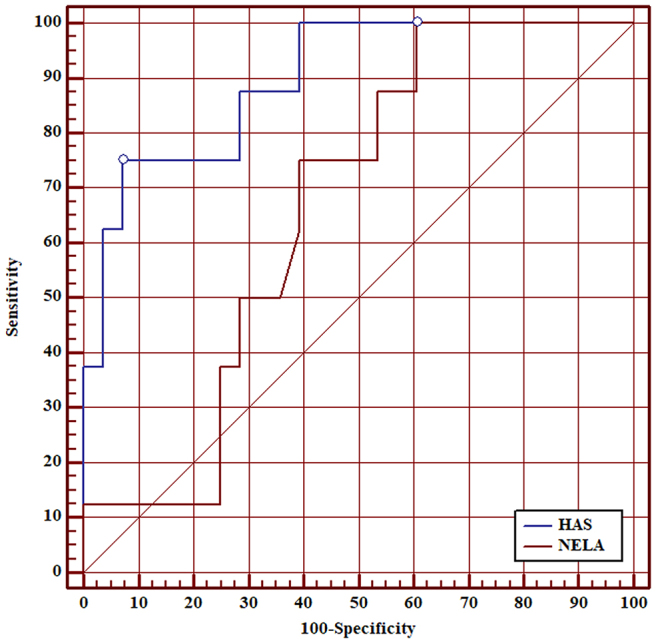
ROC curve for comparison between HAS score and NELA score in predicting 30-day mortality in patients aged ≥80 years. HAS = Hajibandeh Index, American Society of Anaesthesiologists status, and sarcopenia; NELA = National Emergency Laparotomy Audit; ROC = receiver operating characteristic.

ROC curve analysis showed that AUC of the HAS score for 30-day mortality was 0.90 (95% CI 0.83–0.95). Although the AUC of the HAS score was higher than the AUC of the NELA score for all patients, this was not statistically significant (0.90 vs 0.80, *p*=0.097) (Figure 1). AUC of the HAS score was superior to the NELA score in patients aged ≥50 (0.89 vs 0.75, *p*=0.040) (Figure 2), patients aged ≥60 (0.87 vs 0.69, *p*=0.020) (Figure 3), patients aged ≥70 (0.85 vs 0.67, *p*=0.030) (Figure 4), and patients aged ≥80 (0.90 vs 0.66, *p*<0.001) (Figure 5).

## Discussion

We conducted a retrospective cohort study to externally validate the performance of the HAS model in predicting 30-day mortality in patients undergoing emergency laparotomy. Our analysis included 117 patients, and the results indicated that the AUC of the HAS model for predicting 30-day mortality was 0.90 (95% CI 0.83–0.95). This high AUC demonstrates excellent discriminatory ability. Moreover, the HAS score outperformed the NELA score across various age groups: patients aged ≥50, ≥60, ≥70, and ≥80 years.

The findings of the present study align with those of Hajibandeh *et al*,^2^ who initially developed the HAS model. Their research also showed that the HAS model exhibited outstanding discrimination in predicting 30-day mortality. Furthermore, consistent with Linganathan *et al*,^6^ our study found that the HAS model’s performance surpassed that of the NELA score. This superior performance of the HAS model can be attributed to several factors.

First, the HAS model incorporates comprehensive measures of the patient’s physical status, such as comorbidities, frailty and physiological reserve, which are assessed through the ASA status and the presence of sarcopenia.^2^ These factors are critical as they provide a holistic view of the patient’s overall health and ability to withstand surgical stress. Additionally, the HAS model considers the severity of abdominal pathology using the HI. The HI is associated strongly with peritoneal contamination, tissue necrosis and intestinal ischemia.^5^

Despite the promising results, it is essential to acknowledge the limitations of the current study. One of the main limitations is the relatively small sample size of 117 patients. This limitation raises concerns about the potential for a type 2 error. Another limitation is the exclusion of 15 patients due to unavailable perioperative data. This exclusion, combined with the small sample size, further exacerbates the risk of type 2 error. Moreover, the study was conducted at a single centre, which limits the generalisability of the findings to other settings. The retrospective nature of the study would subject the results to inevitable risk of selection bias.

To address these limitations and strengthen the evidence supporting the HAS model, future research should focus on larger, multicentre studies with prospective designs. These studies would provide a more robust assessment of the HAS model’s performance across diverse patient populations and clinical settings. Additionally, prospective studies can help ensure more comprehensive and accurate data collection, reducing the risk of biases associated with retrospective analyses.

## Conclusion

The results of the current study support the external validity of the HAS model in predicting 30-day mortality following emergency laparotomy. Our findings demonstrate that the HAS model has excellent discriminatory ability, outperforming the NELA score across various age groups. However, due to the study’s limitations, including a relatively small sample size, single-centre design and retrospective nature, there is a need for further research. Prospective studies with larger and more diverse patient populations are required to confirm these results and establish the HAS model as a reliable tool for predicting postoperative outcomes in clinical practice.

## References

[1] Parsimonious NELA Risk Calculator. https://data.nela.org.uk/riskcalculator/ (cited February 2025).

[2] Hajibandeh S, Hajibandeh S, Hughes I *et al.* Development and validation of HAS (Hajibandeh index, ASA status, sarcopenia) - a novel model for predicting mortality after emergency laparotomy. *Ann Surg* 2023; **279**: 501–509. 37139796 10.1097/SLA.0000000000005897

[3] Thahir A, Pinto-Lopes R, Madenlidou S *et al.* Mortality risk scoring in emergency general surgery: are we using the best tool? *J Perioper Pract* 2021; **31**: 153–158. 32368947 10.1177/1750458920920133

[4] Barazanchi A, Bhat S, Palmer-Neels K *et al.* Evaluating and improving current risk prediction tools in emergency laparotomy. *J Trauma Acute Care Surg* 2020; **89**: 382–387. 32301890 10.1097/TA.0000000000002745

[5] Hajibandeh S, Hajibandeh S, Waterman J *et al.* Hajibandeh index versus NELA score in predicting mortality following emergency laparotomy: a retrospective cohort study. *Int J Surg* 2022; **102**: 106645. 35533852 10.1016/j.ijsu.2022.106645

[6] Linganathan S, Hughes I, Puthiyakunnel Saji A *et al.* HAS (Hajibandeh index, American society of anesthesiologists status, and sarcopenia) model versus NELA (National Emergency Laparotomy Audit) score in predicting the risk of mortality after emergency laparotomy: a retrospective cohort study. *Cureus* 2023; **15**: e50180. 38077684 10.7759/cureus.50180 PMC10706199

[7] HAS Emergency Laparotomy Mortality Risk Calculator. https://app.airrange.io/#/element/xr3b_E6yLor9R2c8KXViSAeOSK (cited February 2025).

[8] American Society of Anaesthesiologists Committee on Economics. *ASA Physical status classification system*. https://www.asahq.org/standards-and-guidelines/asa-physical-status-classification-system (cited February 2025).

[9] Eugene N, Oliver CM, Bassett MG *et al.* Development and internal validation of a novel risk adjustment model for adult patients undergoing emergency laparotomy surgery: the National Emergency Laparotomy Audit risk model. *Br J Anaesth* 2018; **121**: 739–748. 30236236 10.1016/j.bja.2018.06.026

